# Diffusion weighted imaging-based differentiation of arteritic and non-arteritic anterior ischemic optic neuropathy

**DOI:** 10.1186/s12880-025-01780-4

**Published:** 2025-06-19

**Authors:** Charlotte Pietrock, Theresia Knoche, Sophia Meidinger, Victor Wenzel, Konrad Neumann, Eberhard Siebert, Leon Alexander Danyel

**Affiliations:** 1 Department of Neurology with Experimental Neurology, Charité – Universitätsmedizin Berlin, Augustenburger Platz 1, 13353 Berlin, Germany; 2 Institute of Biometry and Clinical Epidemiology, Charité – Universitätsmedizin Berlin, Charitéplatz 1, 10117 Berlin, Germany; 3 Institute of Neuroradiology, Charité – Universitätsmedizin Berlin, Charitéplatz 1, 10117 Berlin, Germany

**Keywords:** Giant cell arteritis, Optic neuropathy, Diffusion-weighted imaging, Magnetic resonance imaging

## Abstract

**Purpose:**

To assess the utility of DWI-MRI to differentiate arteritic (A-AION) from non-arteritic (NA-AION) ischemic optic neuropathy.

**Methods:**

This bicentric cohort-study evaluated 3T DWI-MRI scans performed within 10 days after onset of AION in patients treated between 2014 and 2024 at two tertiary care centers. DWI was first assessed for the presence of restricted diffusion within the optic nerve. Quantitative apparent diffusion coefficient (ADC) evaluation was performed by placing a region of interest (ROI) within the affected optic nerve. Qualitative and quantitative DWI assessments were compared between A-AION and NA-AION patients.

**Results:**

Twenty A-AION patients (75.7 ± 6.8 years; 16 [80.0%] female) and 59 NA-AION patients (64.6 ± 10.7 years; 22 [37.3%] female) with a total of 82 (A-AION: 23; NA-AION: 59) DWI-MRI scans were included in the study. Restricted diffusion on ADC was significantly more frequent in A-AION, when compared to NA-AION (82.6% vs. 42.4%; *p* = 0.001). Corresponding sensitivity, specificity, positive and negative predictive value of qualitative ADC assessment for the identification of A-AION were 0.83, 0.58, 0.43 and 0.89. Quantitative ADC analysis revealed significantly lower values in optic nerves affected by A-AION (ADC: 448.0 ± 256.2 × 10^− 6^ mm^2^/s vs. 671.5 ± 174.9 × 10^− 6^ mm^2^/s, *p* = 0.002).

**Conclusion:**

Restricted diffusion of the optic nerve is more frequent in A-AION and associated with lower optic nerve ADC values, when compared to NA-AION. Prospective studies are required to further explore the potential of DWI in discerning arteritic from non-arteritic AION.

## Introduction

Anterior ischemic optic neuropathy (AION) is an acute disorder of the optic nerve head caused by a vascular insufficiency of the short posterior ciliary arteries resulting in sudden, painless monocular vision loss [1–3]. It is the most common acute optic neuropathy in the elderly and is generally classified into two types: non-arteritic (NA-AION) and arteritic (A-AION) [1–4].

NA-AION constitutes the majority (approx. 90–94%) of all AION cases [3, 5]. The estimated annual incidence is 2.3–10.2 per 100 000 people over 50 years of age, with only occasional occurrences in younger patients [6–8]. It affects both sexes equally. Clinically, NA-AION presents with vision loss, ranging from visual-field defects to complete monocular blindness, accompanied by a relative afferent pupillary defect and optic disc edema [4, 9]. Fundoscopic examination reveals a small cup to disc ratio also known as a “disc at risk” [4, 9]. Visual impairment is generally permanent and the risk of second eye involvement ranges between 12 and 25% over a 5-year period [2, 4, 10]. Aside from controlling modifiable vascular risk factors, no established therapeutic measures exist to date [4, 9].

A-AION almost never manifests before the age of 50 and is more prevalent in women [2, 4, 9, 11–13]. It has an annual incidence rate of 0.4 per 100 000 people over 50 years [7]. A-AION is primarily associated with giant cell arteritis (GCA) and more rarely with other types of autoimmune or infectious vasculitis [2, 4, 14]. Visual loss in A-AION is typically more severe than in NA-AION and, if left untreated, the risk of second-eye involvement is at least 50% within weeks [4, 9]. About 75% of patients experience systemic symptoms (e.g., weight loss, abnormal temporal artery, jaw or tongue claudication, temporal headache, scalp tenderness, myalgia, morning stiffness, shoulder or neck pain) prior or concomitant to the visual symptoms [4, 15]. Fundoscopic findings include optic nerve pallor and edema [4]. Additionally, increased erythrocyte sedimentation rate and C-reactive protein level, a circumferential hypoechogenic vessel wall thickening (“halo sign”) of the superficial temporal artery on ultrasonography, fluorescein angiography or temporal artery biopsy can aid the diagnostic process, but should not delay therapy [2, 4, 9, 16]. A-AION is considered a neuro-ophthalmological emergency requiring immediate high-dose systemic corticosteroid therapy to preserve the remaining vision and prevent involvement of the second eye [2–4, 9, 13, 15].

Despite these epidemiological, symptom-related and paraclinical differences, the differentiation between NA-AION and A-AION can be challenging in clinical practice. On the one hand, cortisone pulse therapy must be started as soon as possible in the case of A-AION to prevent further visual deterioration or involvement of the remaining eye. On the other hand, unnecessary cortisone therapy in the context of NA-AION can lead to severe corticosteroid-associated adverse effects, especially because dosing is tapered over many months. Once corticosteroids have been administered, their anti-inflammatory properties progressively render the diagnosis of GCA more difficult or even impossible. Rapid etiological assessment is therefore of central importance in the medical care of AION patients.

Diffusion-weighted magnetic resonance imaging (DWI-MRI) is a precise and non-invasive method for detecting cerebral ischemia by analyzing the apparent diffusion coefficient (ADC) of water within brain tissue [14, 17]. Many case reports identified DWI-abnormalities in the optic nerve of patients with NA-AION [14]. Recent findings demonstrated that DWI-MRI can effectively identify anterior and posterior optic nerve ischemia in patients with GCA [14]. Mournet et al. and Adesina et al. successfully applied DWI-MRI to distinguish ischemic from inflammatory optic neuropathies [18, 19]. In a subanalysis, Mournet et al. provided preliminary results that DWI-MRI may be used to discriminate NA-AION and A-AION patients [18]. However, the evidence is limited to a retrospective cohort analysis with a small sample size of AION patients [18].

Consequently, the purpose of our study was to assess the utility of DWI-MRI in the differentiation of NA-AION and A-AION patients.

## Patients and methods

In this retrospective cohort study, DWI-MRI scans of arteritic and non-arteritic AION patients were evaluated for the presence of restricted diffusion of the optic nerve. Study approval was granted by the ethics committee of the Charité – Universitätsmedizin Berlin (registration number: EA1/235/23). The committee determined that informed consent was not required for this retrospective cohort study. The study was conducted in accordance with the relevant guidelines and/or regulations including the Declaration of Helsinki.

### Patient selection

Consecutive AION patients treated at the two tertiary care centers (Charité Campus Virchow-Klinikum and Charité Campus Benjamin Franklin) between January 2014 and April 2024 with available 3T DWI-MRI performed within 10 days after onset of visual impairment were included in the study. The patient selection process was as follows:

First, a systematic database inquiry was performed to identify potential AION patients with available DWI-MRI based on codes of the International Classification of Diseases (ICD-10; H47.0 and M31.5-6) and the German Operation and Procedure Classification System (OPS; 3–800 and 3–820). The medical records of identified patients were screened for (1) diagnosis of AION and (2) available 3T DWI-MRI performed within 10 days from onset of visual impairment. Diagnosis of AION was based on (1) clinical presentation of sudden onset of visual impairment, (2) ophthalmological assessment (revealing optic disc edema, relative afferent pupillary defect and visual field defects) and (3) exclusion of competing diagnoses by brain MRI. AION patient records were then reviewed for demographic data, technical information regarding the MRI scanner and protocol, possible corticosteroid therapy, as well as information relevant to a diagnosis of GCA according to the 2022 American College of Rheumatology/EULAR classification criteria for GCA [20]. A diagnosis of A-AION was established in AION patients who, after review of the interdisciplinary diagnostic evaluation for giant cell arteritis, reached a sum score of ≥ 6 according to the clinical, laboratory, imaging and biopsy criteria of the 2022 American College of Rheumatology/EULAR classification criteria for giant cell arteritis. NA-AION patients showed no evidence of medium or large-vessel vasculitis and did not meet the aforementioned diagnostic criteria.

### Imaging acquisition and analysis

DWI-MRI was performed during routine diagnostic work-up of AION patients. A 3T scanner with a 20-channel head coil (Skyra, Siemens, Erlangen, Germany), a 3T scanner with a 16-channel head coil (Vida, Siemens, Erlangen, Germany) and a 3T scanner with a 32-channel head coil (Trio, Siemens, Erlangen, Germany) were used to acquire DWI sequences. These consisted of either DWI-EPI, DWI-RESOLVE or reconstructed DWI images from diffusion tensor imaging (DTI) sequences with slice thicknesses ranging from 2 mm to 4 mm. Image evaluation was performed by a board-certified neuroradiologist (> 15 years of experience in MR imaging), who was presented randomized sets of imaging studies for analysis. The neuroradiologist was informed about the side of the affected optic nerve for qualitative and quantitative analysis, but blinded for all clinical data and etiology of AION (arteritic or non-arteritic).

Image analysis was performed using the Merlin Diagnostic Workcenter (Phoenix-PACS GmbH, Freiburg, Germany). Firstly, axial DWI b1000 images were scrutinized for pathologically increased signal within the optic nerve head and the evaluable part of the intraorbital portion of the optic nerve. The corresponding area on the axial ADC map was evaluated qualitatively for the presence or absence of diffusion restriction by comparing it to the signal of normal-appearing temporal lobe white matter, as described by Mournet and colleagues [18]. Restricted diffusion of the optic nerve was rated as present, if the focal ADC values were comparatively lower than those of the temporal lobe. In patients with qualitative diffusion restriction, an oval or round ROI was drawn manually, taking care to include only the area of restriction diffusion within the optic nerve. Both the mean and the lowest ADC ROI-based measurement, as provided by the image analysis software upon placing the ROI, were used for further quantitative analysis. Finally, after a period of 10 months, quantitative ADC analysis was repeated, methodically identical to the first assessment.

### Statistical analysis

Statistical analyses were performed using IBM SPSS Statistics software (IBM SPSS Statistics for Windows, Version 25.0. Armonk, NY: IBM Corp.) and GraphPad Prism (Version 8.0.0 for Windows, GraphPad Software, San Diego, CA, USA). Descriptive statistics are presented as the mean ± standard deviation or median with indication of the first and third quartile. The primary null hypothesis was tested at a two-sided significance level of α = 0.05. All other tests were considered exploratory, and no adjustments for multiple comparisons were made.

The primary analysis compared the presence of restricted diffusion of the optic nerve on ADC maps between the NA-AION and A-AION group using Pearson’s chi-square test. Additionally, standard diagnostic performance metrics, including sensitivity, specificity, and positive and negative predictive values, were calculated. Binary logistic regression analysis was conducted to assess the impact of AION subtype (A-AION or NA-AION), age and sex on the presence of restricted diffusion of the optic nerve on ADC maps. Sex and age were included in the multiple regression model due to notable differences between NA-AION and A-AION patients in these characteristics.

We compared the lowest and mean ADC values within the ROI of NA-AION and A-AION patients using separate independent samples t-tests. Corresponding receiver operating characteristic curves (ROC) were constructed. Linear regression analysis was performed with ADC value measurements as the dependent variable and age, sex and MRI scanner type as independent variables. The reproducibility of quantitative ADC analysis was evaluated using the Bland-Altman method by comparing the primary assessment with a repeated assessment after 10 months. In addition, the intraclass correlation coefficient (ICC) was calculated to assess inter-rater reliability between the two assessments.

Finally, a subanalysis of both quantitative and qualitative ADC measurements was performed in patients with NA-AION and A-AION using DWI-MRI obtained within 5 days of the onset of visual impairment, with Pearson’s chi-square tests and independent-samples t-tests were applied for statistical evaluation.

## Results

**Fig. 1:**
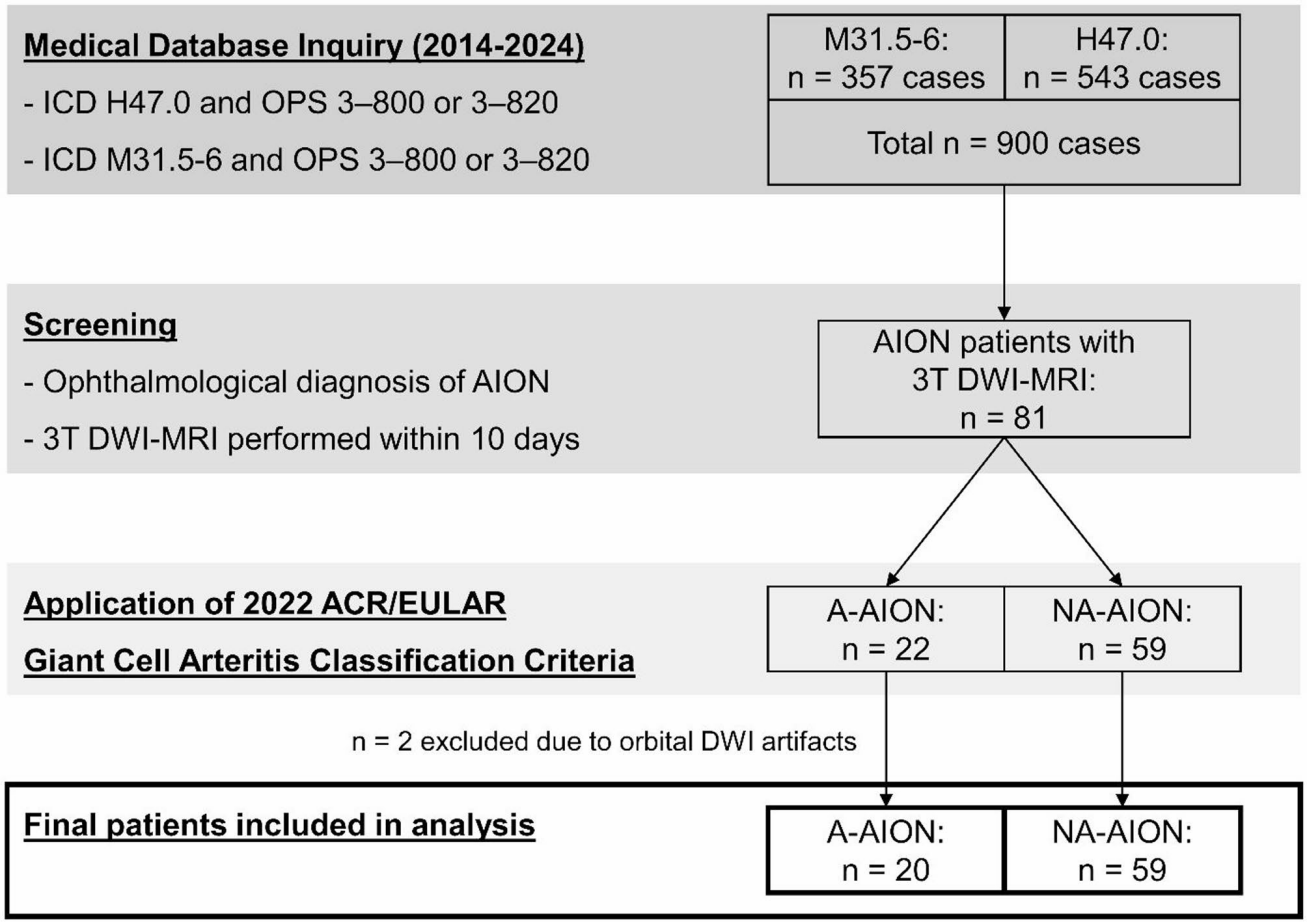
Flow diagram of the patient selection process. Abbreviations: ACR, American College of Rheumatology; AION, anterior ischemic optic neuropathy; A-AION, arteritic anterior ischemic optic neuropathy; DWI-MRI, diffusion-weighted magnetic resonance imaging; ICD, International Statistical Classification of Diseases and Related Health Problems; NA-AION, non-arteritic anterior ischemic optic neuropathy

Twenty A-AION patients (75.7 ± 6.8 years; 16 [80.0%] female) and 59 NA-AION patients (64.6 ± 10.7 years; 22 [37.3%] female) were included in the study. Figure 1 illustrates the patient selection process.

In A-AION, optic nerve ischemia affected the left side in 10 (50.0%), the right side in 7 (35.0%) and both optic nerves in 3 (15.0%) patients. NA-AION was left-sided in 29 (49.2%) and right-sided in 30 (50.8%) with no cases of bilateral affection observed. Table 1 details clinical characteristics and laboratory findings, as well as temporal artery biopsy and superficial temporal artery ultrasound results in A-AION and NA-AION patients. Corticosteroid therapy was administered in 95% of A-AION patients (*n* = 19; iv pulse glucocorticoids in all but one case, who received oral glucocorticoids) and 18.6% of NA-AION patients (*n* = 11; iv pulse glucocorticoids in all but one case) before MRI examination.

**Table 1:** Clinical characteristics, laboratory findings, temporal artery biopsy and superficial temporal artery ultrasound results in arteritic (A-AION) and non-arteritic anterior ischemic optic neuropathy (NA-AION) patients (adapted from the 2022 American college of rheumatology/eular classification criteria for giant cell arteritis)

	A-AION	NA-AION
	*n* = 20	*n* = 59
Age (years, mean ± SD)	75.7 ± 6.8	64.6 ± 10.7
Female (*n*, %)	16 (80.0%)	22 (37.3%)
**Clinical characteristics**		
Morning stiffness in shoulders/neck (*n*, %)	3 (15.0%)	Not reported
Jaw or tongue claudication (*n*, %)	11 (55.0%)	Not reported
New temporal headache (*n*, %)	14 (70.0%)	Not reported
Scalp tenderness (*n*, %)	5 (25.0%)	Not reported
Abnormal examination of the temporal artery (*n*, %)	9 (45.0%)	Not reported
**Laboratory findings**		
History of ESR ≥ 50 mm/h (*n*, %)	18 (90.0%)	3 (5.1%)
ESR (mm/h; mean ± SD)	60.5 ± 11.0	18.4 ± 18.6
Maximum C-reactive protein ≥ 10 mg/l (*n*, %)	18 (90.0%)	6 (10.2%)
C-reactive protein (mg/l; mean ± SD)	86.6 ± 80.3	10.0 ± 46.1
**Superficial temporal artery ultrasound**		
US performed (*n*, %)	18 (90.0%)	29 (49.2%)
STA Halo (*n*, %)	12/18 (66.7%)	0/29 (0%)
**Temporal artery biopsy**		
TAB performed (*n*, %)	11 (55.0%)	8 (13.6%)
GCA-positive TAB (*n*, %)	7/11 (63.6%)	0/8 (0%)

Abbreviations: A-AION, arteritic anterior ischemic optic neuropathy; NA-AION, non-arteritic anterior ischemic optic neuropathy; ESR, erythrocyte sedimentation rate; US, ultrasound; STA, superficial Temporal artery; TAB, Temporal artery biopsy; GCA, giant cell arteritis

A total of 82 (A-AION: 23; NA-AION: 59) 3T DWI-MRI scans were evaluated. The most common sequence types used were DWI-EPI TRACE and trace DWI from DTI-EPI sequence with slice thicknesses ranging from 2 to 4 mm. Mean time-delay between onset of visual impairment and DWI acquisition was 3.6 ± 1.7 days in A-AION and 4.8 ± 2.3 days in NA-AION patients. Table 2 details DWI-MRI imaging characteristics in AION patients.

**Table 2:** Imaging features of DWI-MRI in arteritic (A-AION) and non-arteritic anterior ischemic optic neuropathy (NA-AION) patients

MRI parameters	A-AION	NA-AION
3T Scans performed	*n* = 23	*n* = 59
Time from onset to DWI-MRI		
0-5d (*n*, %)	21 (91.3%)	35 (59.3%)
6-10d (*n*, %)	2 (8.7%)	21 (35.6%)
Assignment not possible (*n*, %)	0 (0%)	3 (5.1%)
Slice Thickness		
2.0 mm (*n*, %)	0 (0%)	1 (1.7%)
2.5 mm (*n*, %)	14 (60.9%)	30 (50.9%)
3 mm (*n*, %)	9 (39.1%)	27 (45.8%)
4 mm (*n*, %)	0 (0%)	1 (1.7%)
Sequence Type		
DWI-EPI (*n*, %)	19 (82.6%)	52 (88.1%)
DTI (*n*, %)	4 (17.4%)	6 (10.2%)
DWI-RESOLVE (*n*, %)	0 (0%)	1 (1.7%)

Abbreviations: A-AION, arteritic anterior ischemic optic neuropathy; NA-AION, non-arteritic anterior ischemic optic neuropathy; MRI, magnetic resonance imaging; DWI, diffusion-weighted imaging; DTI, diffusion tensor imaging

High signal of the optic disc or intraorbital optic nerve on DWI was present in 45 of 59 NA-AION (76.3%) and 22 of 23 A-AION scans (95.7%). Restricted diffusion on ADC was significantly more frequent in A-AION, when compared to NA-AION (19/23 or 82.6% vs. 25/59 or 42.4% in NA-AION; *p* = 0.001). Corresponding sensitivity, specificity, positive and negative predictive value of qualitative ADC assessment for the identification of A-AION were 0.83 (95% CI 0.61–0.94), 0.58 (95% CI 0.44–0.70), 0.43 (95% CI 0.29–0.59) and 0.89 (95% CI 0.74–0.97). Binary logistic regression analysis demonstrated a significantly higher prevalence of restricted diffusion of the optic nerve on ADC maps in the A-AION group compared to the NA-AION group, with an odds ratio of 6.44 (95% CI: 1.67–24.88, *p* = 0.007). Sex and age were not associated with the presence of restricted diffusion of the optic nerve (sex: OR = 0.71, 95% CI: 0.26–1.96, *p* = 0.51; age: OR = 1.02, 95% CI: 0.97–1.06, *p* = 0.54).

**Fig. 2:**
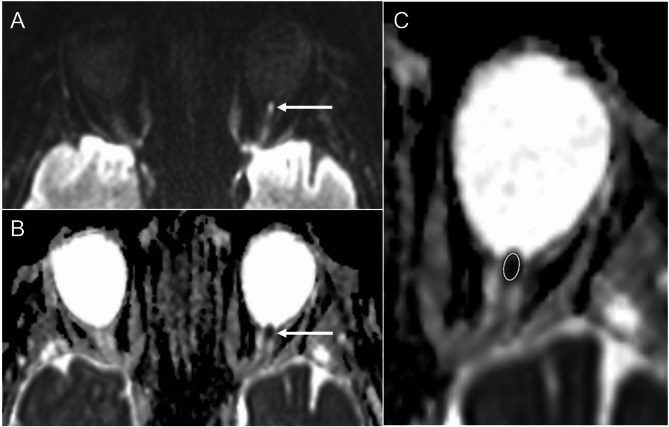
Qualitative and quantitative ADC map assessment in a patient with left anterior ischemic optic neuropathy. Hyperintensity of the left optic nerve head is visible on DWI (**A**). Corresponding reduction of the ADC inferior to the signal of the white matter of the temporal lobe is visible (qualitative assessment, (**B**). For the quantitative assessment, a region of interest (ROI) presenting the lowest signal on ADC maps was placed within the optic nerve head/intraorbital portion of the optic nerve (**C**)

Exploratory quantitative ADC analysis revealed significantly lower values in optic nerves affected by A-AION, if the lowest ADC values within the ROIs were considered (ADC: 448.0 ± 256.2 × 10^− 6^ mm^2^/s vs. 671.5 ± 174.9 × 10^− 6^ mm^2^/s, *p* = 0.002). Similarly, significant differences were observed when mean ADC values were calculated from the ROIs (ADC: 730.0 ± 235.9 × 10 − 6 mm2/s vs. 865.2 ± 159.2 × 10 − 6 mm2/s, *p* = 0.044). ROI size was 3.02 ± 1.60 mm^2^ (range 1.40–6.70 mm^2^) in NA-AION, compared to 4.16 ± 2.03 mm^2^ (range 1.90–7.80 mm^2^) in A-AION. Linear regression analysis did not reveal a significant association between mean ADC value measurement and age (ß = 0.023, *p* = 0.896), sex (ß = 0.045, *p* = 0.796) or scanner type (ß = -0.096, *p* = 0.560). The overall model was not significant (R^2^ = 0.010, *p* = 0.939). Similarly, no significant associations were observed between lowest ADC value measurements and age (ß = 0.009, *p* = 0.960), sex (ß = 0.226, *p* = 0.183) or scanner type (ß = -0.192, *p* = 0.233). Again, the overall model was not significant (R^2^ = 0.087, *p* = 0.321). Table 3 details quantitative ADC measurements in DWI of AION patients. An exemplary qualitative and quantitative ADC measurement in an AION patient is illustrated in Fig. 2.

**Table 3:** Quantitative ADC assessment in diffusion-weighted imaging (DWI) of non-arteritic (NA-AION) and arteritic (A-AION) anterior ischemic optic neuropathy

Quantitative ADC	DWI performed within 10 days		p-value	DWI performed within 5 days		p-value
[× 10^− 6^ mm^2^/s]	A-AION	NA-AION		A-AION	NA-AION	
**Optic nerve ADC**_**Low**_						
Mean (± SD)	448.0 (± 256.2)	671.5 (± 174.9)	0.002	421.6 (± 249.4)	700.7 (± 174.5)	< 0.001
Median (Q1; Q3)	445.0 (268.3; 673.5)	650.5 (550.0; 806.5)		397.0 (224.8; 615.0)	712.5 (628.3; 816.3)	
**Optic nerve ADC**_**Mean**_						
Mean (± SD)	730.0 (± 235.9)	865.2 (± 159.2)	0.044	698.6 (± 229.0)	870.4 (± 174.0)	0.021
Median (Q1; Q3)	811.0 (517.8; 886.8)	857.0 (778.5; 962.0)		784.0 (489.3; 885.3)	857.0 (779.0; 955.8)	

Abbreviations: A-AION, arteritic anterior ischemic optic neuropathy; NA-AION, non-arteritic anterior ischemic optic neuropathy; DWI, diffusion-weighted imaging; ADC, apparent diffusion coefficient

**Fig. 3:**
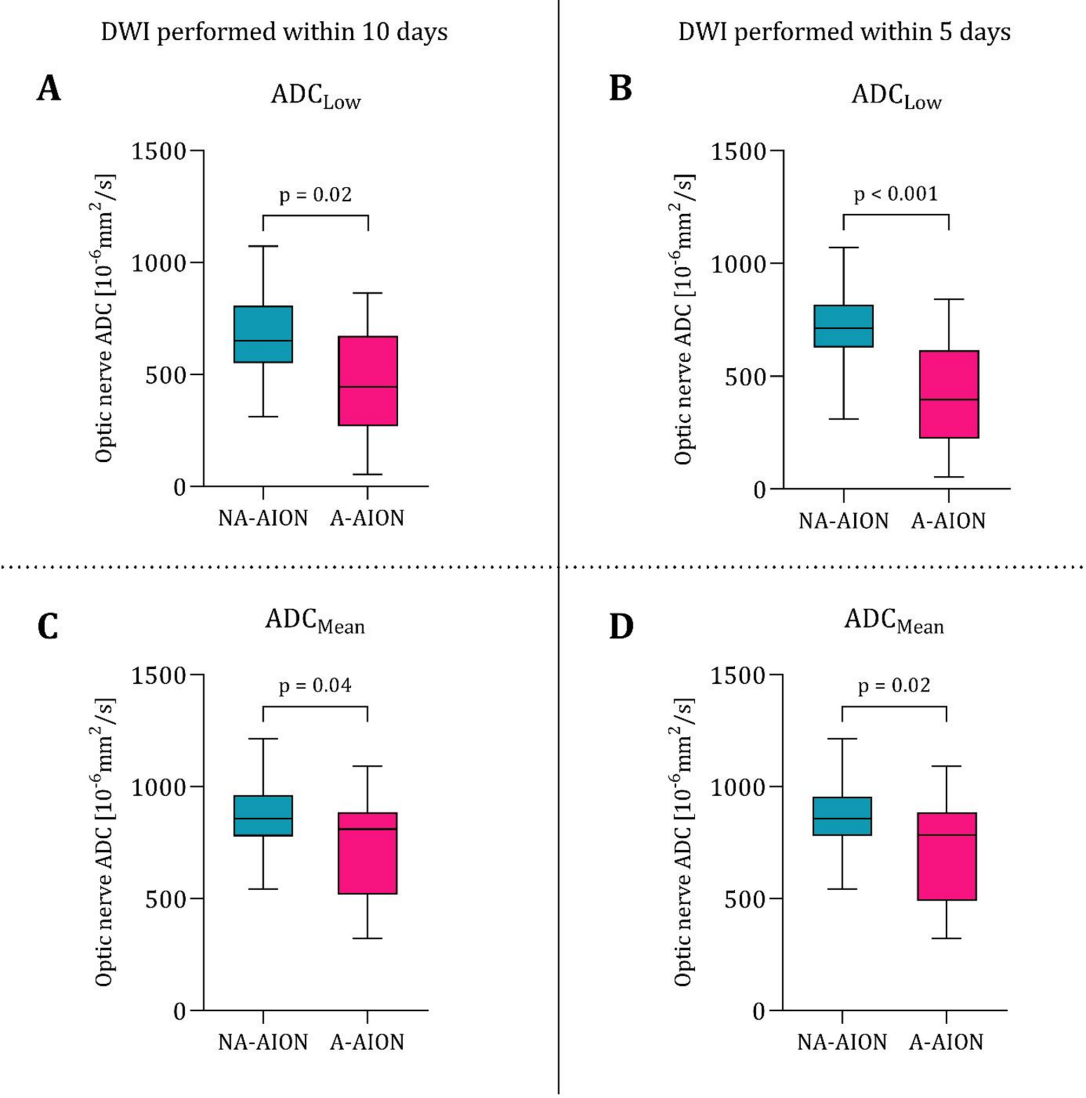
Box plots illustrating the quantitative optic nerve apparent diffusion coefficient (ADC) assessment in arteritic (A-AION) and non-arteritic (NA-AION) ischemic optic neuropathy for diffusion-weighted imaging performed within 10 days (**A**, **C**) and 5 days (**B**, **D**). Measurements were based on the lowest ADC measured (ADC_Low_: **A**, **B**) or the calculated mean ADC values within the ROIs (ADC_Mean_: **C**, **D**)

**Fig. 4:**
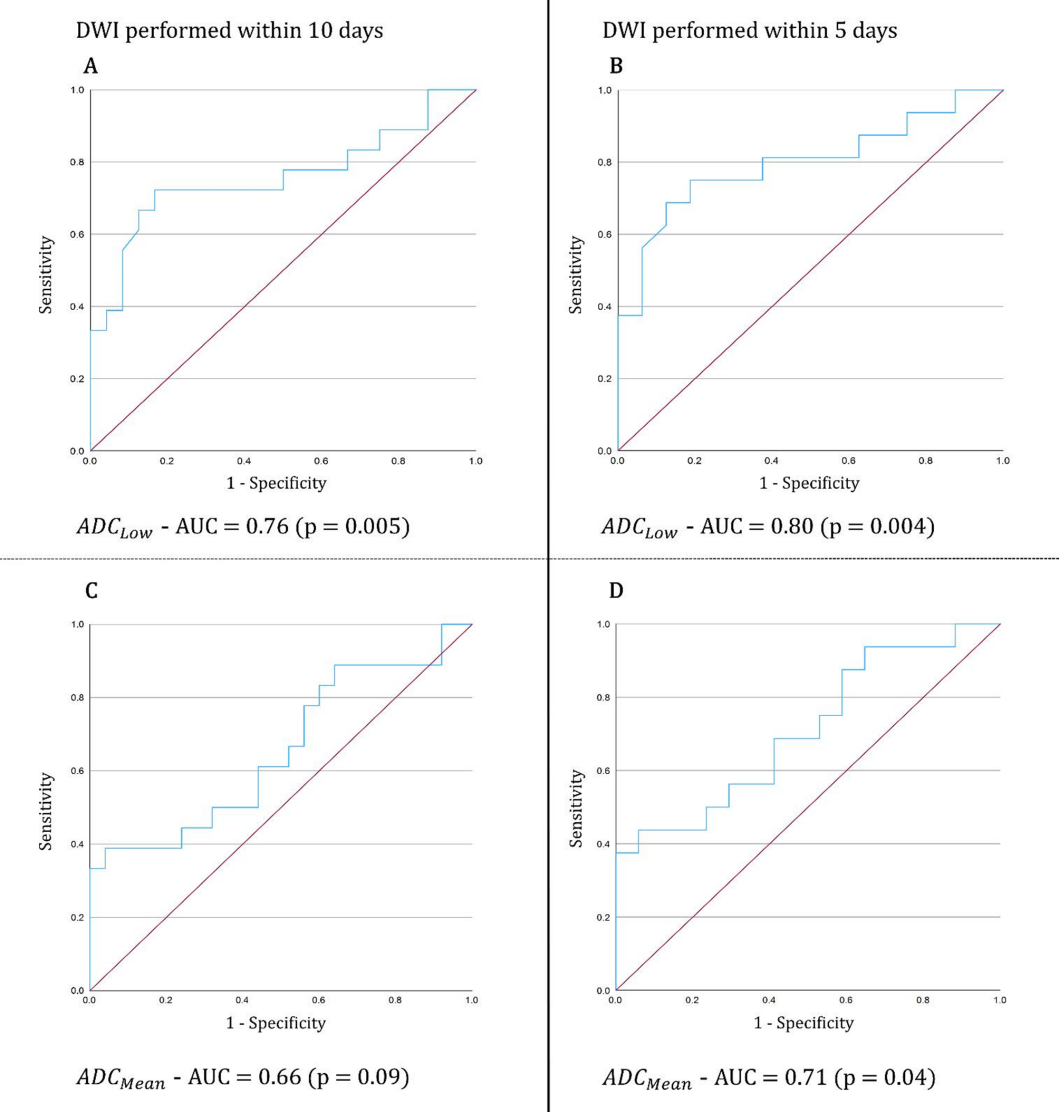
Receiver Operator Characteristic (ROC) curves of quantitative apparent diffusion coefficient (ADC) assessment for the diagnostic differentiation of arteritic (A-AION) from non-arteritic (NA-AION) anterior ischemic optic neuropathy. Separate ROC curves were constructed for diffusion-weighted-imaging performed within 10 days (**A**, **C**) and 5 days (**B**, **D**), as well as ADC measurement approach (ADC_Low_ (**A**, **B**): lowest ADC value measured within the region of interest; ADC_Mean_ (**C**, **D**): calculated mean ADC value within the region of interest). AUC, area under curve

In a subanalysis of DWI performed within 5 days after onset of visual impairment, restricted diffusion of the optic nerve in A-AION remained significantly more frequent, when compared to NA-AION patients (17/21 or 81.0% vs. 17/35 or 48.6%; *p* = 0.016). Similarly, quantitative ADC assessment confirmed lower values in optic nerves affected by A-AION, both for the lowest ADC measured (ADC: 421.6 ± 249.4 × 10^− 6^ mm^2^/s vs. 700.7 ± 174.5 × 10^− 6^ mm^2^/s, *p* < 0.001) and for the calculated mean ADC within the ROI (ADC: 698.6 ± 229.0 × 10^− 6^ mm^2^/s vs. 870.4 ± 174.0 × 10^− 6^ mm^2^/s, *p* = 0.021). An ADC threshold of 467 mm^2^/s or lower (lowest ADC measured within the ROI) of DWI performed within 5 days after AION onset yielded a specificity of 94%, while retaining a sensitivity of 56% to discern A-AION from NA-AION in patients with presence of (qualitative) restricted diffusion of the optic nerve. Box plots and ROC curves for all quantitative assessments are presented in Figs. 3 and 4.

**Fig. 5:**
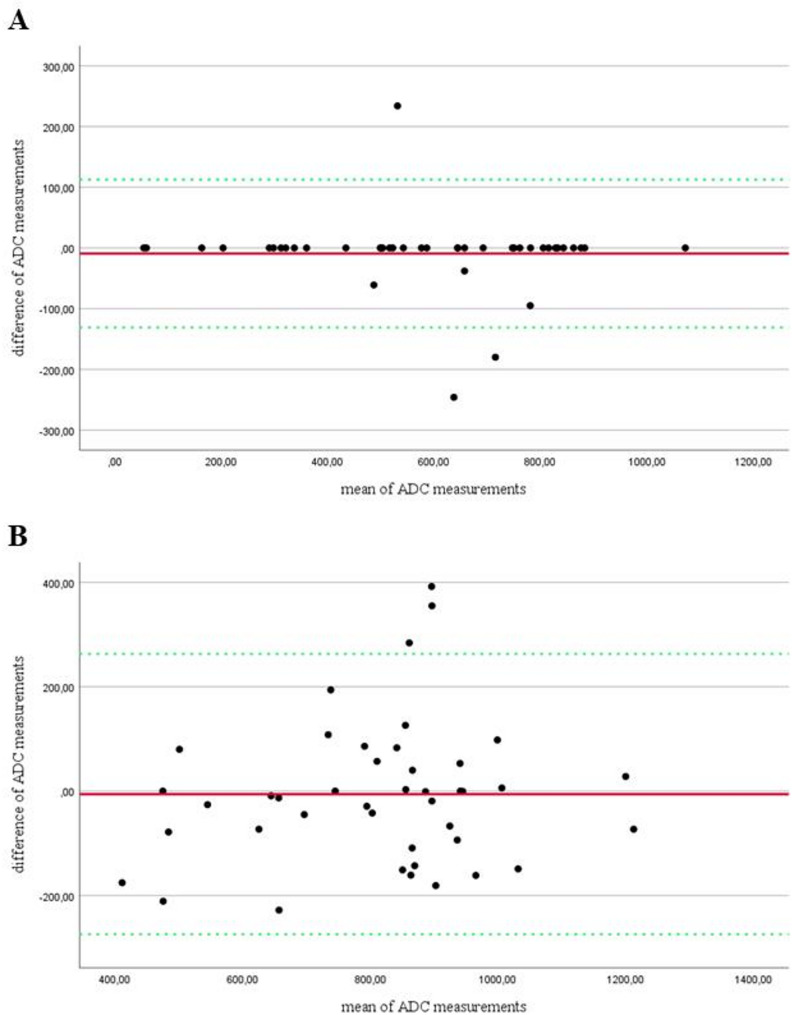
Bland-Altman plots. To assess the quality of agreement between the first and repeated quantitative ADC analysis performed by the neuroradiologist, scatter plots were constructed, plotting the differences between two corresponding ADC value measurements ([10 − 6 mm2/s], y-axis) against their averages ([10 − 6 mm2/s], x-axis). Separate plots are shown for the two measurement approaches used (**A**: lowest ADC value within the ROI; **B**: calculated mean ADC value of the ROI). No systematic bias was observed for both approaches as indicated by the red line (mean absolute difference). The lowest ADC value measurement approach (**A**) yielded better overall agreement as indicated by narrower 95% limits of agreement (green dotted lines), when compared to the calculated mean ADC measurement (**B**). As variations in manual ROI placement occur between the first and repeated assessment, mean ADC value measurements within the ROI (**B**) differ due to variations in voxel composition. Lowest ADC value measurements (**A**) are less susceptible to variations in ROI placement and will generate constant ADC values, given the corresponding voxel remains within the ROI and no voxel with lower ADC is introduced

Comparison of the primary and repeated quantitative ADC assessment after 10 months indicated “excellent” intra-rater reliability for lowest ADC measurements (ICC = 0.98 [0.97–0.99]) and “good” intra-rater reliability for the calculation of mean ADC values within the ROIs (ICC = 0.86 [0.74–0.93]). In addition, Bland-Altman plots were used to assess the quality of agreement between the first and repeated quantitative ADC analysis (Fig. 5).

## Discussion

Our systematic investigation of DWI in AION indicates that restricted diffusion of the optic nerve is visible on 3T MRI performed within 10 days after onset of visual impairment. Our findings confirm recent observations of optic nerve signal alterations on DWI in AION: In their 2022 study, Mournet et al. found restricted diffusion of the optic disk and the optic nerve in early DWI performed within 5 days after onset, with a possible application for the differentiation of AION from optic neuritis [18]. The authors argued that ADC signal evolution in AION may be comparable to changes observed in ischemic stroke [21, 22]. This observation is supported by the fact that retinal ganglion cells, which ultimately form the optic nerve, develop from the diencephalon and have metabolic properties comparable to neurons.

Interestingly, a subanalysis by Mournet et al. asserted differences in qualitative and quantitative ADC assessments between NA-AION and A-AION patients, but the overall number of A-AION patients was lower and no information with regards to the time delay between clinical onset and DWI were provided for this group [18]. Here we contribute further evidence for the utility of DWI to discern A-AION from NA-AION as qualitative restricted diffusion of the optic nerve on ADC map was significantly more frequent in A-AION, when compared to NA-AION. Additionally, the results of our quantitative ADC-analysis imply that ADC cut-off values may be utilized to identify A-AION patients.

The deviating signal pattern observed in DWI may be due to distinct differences in the pathophysiology of NA-AION and A-AION: While the exact cause of NA-AION is not yet fully understood, available evidence implies a transient hypoperfusion or non-perfusion of the optic nerve as a characteristic feature [1–4, 9]. NA-AION is considered a multifactorial disorder in which various risk factors, e.g. arterial hypertension, diabetes mellitus, hyperlipidemia, systemic atherosclerosis, sleep apnea, smoking, increased intraocular pressure or chronic optic disc edema predispose to its development [1–4, 9]. Precipitating risk factors, especially nocturnal arterial hypotension, cause a decrease of perfusion pressure below the critical autoregulatory range level and act as the final catalyst in the onset of NA-AION [1–4]. Embolic or other forms of vessel occlusion appear to play only an insignificant role in its pathogenesis [3]. In contrast, A-AION in GCA is caused by a T-cell mediated vasculitis of the posterior ciliary arteries and/or the ophthalmic artery [2, 9, 14]. Here, vessel wall inflammation leads to a persisting thrombotic occlusion and subsequent infarction of the optic nerve head [2]. It is therefore plausible that A-AION causes more severe optic nerve ischemia and cytotoxic oedema, which in turn could explain the more pronounced ADC reduction observed. Interestingly, signal differences in DWI of A-AION patients were detectable, although proportionally more A-AION patients received corticosteroid therapy before undergoing MRI. Since therapy was initiated after onset of AION, irreversible ischemic damage and cytotoxic oedema of the optic nerve had already occurred. Normally, glucocorticoid therapy in A-AION does not substantially improve visual acuity of the affected eye, but is administered to protect the remaining eye from ischemic complications [4]. Early DWI may therefore allow for a differentiation of A-AION from NA-AION, even in patients under glucocorticoid therapy.

As our results confirm the positive preliminary findings of Mournet et al., planning and implementing prospective studies which employ standardized DWI protocols in AION patients is justified [18]. Possible sequence designs could include small field-of-view DWI, non-EPI DWI and fast spin-echo radial acquisition DWI sequence designs, which may help to mitigate motion artifacts of the eye, as well as signal loss and distortion due to susceptibility artifacts caused by magnetic field inhomogeneities [23–26]. Additionally, specialized sequence protocols will have to be tested for reproducibility of ADC estimates across different MRI scanner models, as ADC quantification is affected by DWI acquisition parameters and the imaging platform used [27, 28].

Our study has several limitations, mainly due to its retrospective and cross-sectional design. The patient selection process was based on a medical database inquiry utilizing ICD-10 and OPS, which may have omitted AION patients in cases of miscoding. Additionally, diagnosis of giant cell arteritis in A-AION was based on the 2022 American College of Rheumatology/EULAR classification criteria and did not necessarily require positive temporal artery biopsy. As acquired DWI was derived from two centers, varying scanners, receive coils, and sequences were used for image generation. All DWI scans evaluated in our study constituted “routine” brain MRI, which was not optimized for the visualization of the optic nerve and orbit. It is important to note, that due to the small size of the volume of interest, partial volume averaging inevitably affects quantitative ADC measurements. This also applies to the phenomenon of partial volume averaging across slices, given the non-strictly axial course of the nerve and in case of the optic nerve head being displayed partially on two adjacent slices. As acquired DWI scans were assessed by a single rater, we were not able to evaluate interrater reliability. However, in their study, Mournet et al. found good interrater agreement for the detection of restricted diffusion of the optic nerve and optic disc, as well as good to excellent intraclass correlation for the quantitative assessment of ADC [18]. Our rater was blinded to all clinical data related to a diagnosis of GCA in order to avoid assessment bias.

## Conclusion

In summary, our investigation indicates that restricted diffusion of the optic nerve is more frequent in A-AION and associated with lower ADC values, when compared to NA-AION. Prospective studies employing optimized orbital imaging protocols are justified to further explore the potential of DWI in discerning arteritic from non-arteritic AION.

## Data Availability

The data that support the findings of this study are not openly available due to reasons of sensitivity and are available from the corresponding author upon reasonable request.
